# Is a combination of varenicline and nicotine patch more effective in helping smokers quit than varenicline alone? A randomised controlled trial

**DOI:** 10.1186/1741-7015-11-140

**Published:** 2013-05-29

**Authors:** Peter Hajek, Katie Myers Smith, Al-Rehan Dhanji, Hayden McRobbie

**Affiliations:** 1 UK Centre for Tobacco Control Studies, Wolfson Institute of Preventive Medicine, Barts & The London School of Medicine and Dentistry, Queen Mary, University of London, London Newark Street, London E1 2AT, UK; 2 Barts and The London NHS Trust, London, UK

**Keywords:** Smoking cessation, Varenicline, Nicotine patch

## Abstract

**Background:**

Nicotine replacement therapy (NRT) and varenicline are both effective in helping smokers quit. There is growing interest in combining the two treatments to improve treatment outcomes, but no experimental data exist on whether this is efficacious. This double-blind randomised controlled trial was designed to evaluate whether adding nicotine patches to varenicline improves withdrawal relief and short-term abstinence rates.

**Methods:**

117 participants seeking help to stop smoking were randomly allocated to varenicline plus placebo patch or varenicline plus nicotine patch (15 mg/16 hour). Varenicline use commenced one week prior to the target quit date (TQD), patch use started on the TQD. Ratings of urges to smoke and cigarette withdrawal symptoms were collected weekly over 4 weeks post-TQD. Medication use and smoking status were established at 1, 4 and 12 weeks. Participants lost to follow-up were included as continuing smokers.

**Results:**

92% of participants used both medications during the first week after the TQD. The combination treatment generated no increase in nausea or other adverse effects. It had no overall effect on urges to smoke or on other withdrawal symptoms. The combination treatment did not improve biochemically validated abstinence rates at 1 week and 4 weeks post-TQD (69% vs 59%, p=0.28 and 60% vs 59%, p=0.91, in the nicotine patch and placebo patch arm, respectively), or self reported abstinence rates at 12 weeks (36% vs. 29%, p=0.39, NS).

**Conclusions:**

The efficacy of varenicline was not enhanced by the addition of nicotine patches, although further trials would be useful to exclude the possibility of type II error.

**Trial Registration:**

Clinicaltrials.gov Registration Number: NCT01184664

## Background

Nicotine replacement treatment (NRT) and varenicline are both effective in helping smokers stop smoking [1,2]. Their efficacy however is limited. In routine use, the majority of smokers receiving intensive support and either of these two medications are likely to fail in their attempt to quit [3]. The possibility has been raised that combining these two medications may increase treatment efficacy [4].

In terms of observable effects on smokers, the two medications appear alike although varenicline has antagonist as well as agonist effects. They both seem to achieve their effect on abstinence by alleviating the discomfort of nicotine withdrawal [1,2]. They also make cigarettes smoked while on the medications less rewarding and thus may facilitate extinction. The latter effect has been demonstrated for both varenicline [5-9] and NRT [10,11], although the evidence for the NRT effect seems weak [11].

In terms of neuro-physiological targets, varenicline affects primarily alpha4 beta2 nicotinic acetylcholine receptors (nAChRs) where it has higher affinity than nicotine and so essentially blocks nicotine effects, as well as acting as a partial agonist [12]. Nicotine from NRT acts on nAChRs in a similar manner to nicotine from tobacco smoke, but delivery from NRT is much slower.

It is not clear if the central effects of the two medications are sufficiently different to allow synergy, but if they do differ, their combination could have several beneficial effects. It could in theory improve withdrawal relief and/or assist smokers who may not react much or at all to one of them, but who may be sensitive to the effects of the other. Another putative mechanism concerns the timing of the treatment regimen of the two medications. Varenicline is used for 1–2 weeks prior to quitting, concurrently with smoking. It is possible that from the target quit day (TQD), when nicotine intake from cigarettes ceases, the addition of NRT to replace any effects which nicotine from cigarettes may still have had up to that point, could reduce post-quitting urges to smoke and withdrawal discomfort. It is also possible of course that the targets of the two medications overlap too much for their combination to provide any additional benefit, or that the receptor occupancy provided by varenicline blocks any additional effects of NRT.

Such hypotheses have not been experimentally evaluated so far. One observational study from an in-patient smoking cessation facility found no differences in outcome between a cohort of patients receiving various combinations of NRT products plus varenicline and a cohort receiving various combinations of NRT products alone or with bupropion [4]. The results need to be interpreted with caution as the groups were self-selected and the various medication combinations were not systematic. However, the study provides reassurance that the combination of NRT and varenicline is safe and well tolerated.

The present trial was designed to compare varenicline alone with a combination of varenicline and nicotine patch in effects on urges to smoke, and abstinence for up to four weeks. This was a proof-of-concept trial, in that a positive finding would provide a justification for a long-term outcome trial, while a negative finding would indicate a more definitive result as lack of efficacy during a period in which the combination is expected to have its main effect would be unlikely to translate into a significant effect later on, unless type II error was present.

We included placebo patch to control for participants’ and staff expectations. We did not include study arms using placebo varenicline. Varenicline is superior to nicotine patches across the time period of our study [8,13,14]. Finding patches plus varenicline more effective than patches plus placebo could just reflect the difference between the two medications rather than any effect of their combination. The provision of double placebo to smokers seeking treatment also poses ethical issues. Finding varenicline plus patches more effective than varenicline plus placebo on the other hand would imply beneficial effects of the combination.

The study was authorized by the UK Medicines and Healthcare Products Regulatory Agency (MHRA) and by the National Research Ethics Service (reference 10/H0709/85).

## Methods

### Design overview

#### Objectives

A double-blind randomised controlled trial was conducted to determine if combining NRT and varenicline provides better withdrawal and craving relief and higher abstinence rates than varenicline alone.

### Setting and participants

Smokers seeking treatment were recruited in April 2011 through local advertisments. Volunteers were included if they were aged 18 and over, were not breastfeeding or pregnant, and had no current psychiatric or other serious illness.

Participants attended standard weekly support sessions following withdrawal-oriented treatment protocol [15] as provided by the NHS Stop-Smoking Service. The trial took place at the Tobacco Dependence Research Unit, Wolfson Institute of Preventive Medicine, Queen Mary University of London.

### Outcomes and follow-up

#### Procedures

Participants attended at baseline, one week prior to TQD, on TQD, and weekly up to week 4 post-TQD. Informed consent was taken at the baseline session. Data were also collected over the phone at 24 hours after the TQD. Participants were contacted for the final follow-up 12 weeks after the TQD. Participants received two payments of £15 at sessions one and four weeks post-TQD.

Participants started varenicline one week before their TQD. On the TQD they were randomised to either a nicotine or placebo patch.

Demographic details, smoking history, and the Fagerstrom Test for Nicotine Dependence (FTND)[16], were collected at baseline. The Mood and Physical Symptoms Scale (MPSS) [17], which assesses tobacco withdrawal symptoms and urges to smoke was completed at all contacts. MPSS asks clients to rate on a 5-point scale how they have been feeling during the past week with regard to depression, irritability, restlessness, hunger, poor concentration and poor sleep at night on a scale ranging from 1=not at all to 5=extremely. The items are analysed separately and also aggregated to give a composite MPSS score. To assess any effect the combination treatment may have on the experience of nausea, we added ‘nausea’ to the scale. The ratings of nausea were not included in the composite MPSS score. A six-point scale is used to rate ‘How much of the time have you felt the urge to smoke in the past week?’ (from ‘not at all (1)’ to ‘all of the time (6)’) and ‘How strong have the urges been?’ (from ‘no urges (1)’ to ‘extremely strong (6)’). The two items are aggregated to give a composite craving score.

At each face-to-face contact, use of trial medication since the previous contact, reports of adverse effects, self-reported smoking status, cigarette consumption since previous contact, and end-expired carbon monoxide levels were assessed. The participants were weighed at baseline and 4 weeks after TQD.

On arrival at the TQD session, participants were sequentially allocated to the study medication by the study staff, using a list that was computer generated by the study statistician (M.S.). Both participants and study staff were blind to treatment allocation. The authors were un-blinded only after the data analysis was completed.

#### Trial medications

##### Varenicline

Commercial supplies were used as per standard labelling (0.5 mg/d for the first 3 days, 1 mg/d on days 4–7, followed by 2 mg/d for the rest of the 12-weeks course).

##### Patches

Nicotine (15 mg/16 hours) and placebo patches were identically packaged. Participants received a box containing a four-week supply (28 patches) on their TQD. We opted for a patch with proven efficacy [18], which avoids sleep disturbance sometimes associated with 24 h patch [1].

#### Sample size

Effective treatments typically generate a difference in MPSS ratings over the first week of abstinence of at least 0.7 compared to control procedures [19], e.g. 1.8 (SD=1) compared to 2.5 (SD=1). As in this case the advantage of the combination over the first week of abstinence may be subtle and even a difference of 0.6 would be worth detecting, 45 participants would be needed in each group (p<0.05, 2-tailed test, power=0.80). To allow for participant attrition, the study aimed to randomise between 110 and 120 participants. This sample size also provides 80% power to detect a clinically meaningful difference between abstinence rates of 50% vs 75% at four weeks (p<0.05, 2-tailed tests).

### Statistical analysis

Differences between the study arms were assessed by analysis of variance for continuous variables and chi-square for categorical variables. The relationship between pre-quit variables and post-quit endpoints was assessed using regression modelling. Differences in urges to smoke at 24 hours and one week were compared using one way ANOVA. Changes in withdrawal symptom ratings from TQD to 24 h and one week were assessed using repeated measures ANOVA. Differences were to be adjusted for all baseline characteristics related to outcome that differed between the two groups. All tests were 2-tailed.

Regarding smoking cessation outcomes, abstinence at 24 hours and 1 week post-TQD was defined as no smoking at all, validated by expired carbon monoxide (CO) reading of <9 ppm. Abstinence at 4 weeks was defined in accordance with the Russell Standard [20], i.e. sustained abstinence since TQD validated by CO reading at all points where CO readings were scheduled (i.e. weeks 1–4 post-TQD), or if a session was missed, self reported sustained abstinence and validation by a CO reading at the next attendance. Up to 5 lapses (single instances of smoking) since the TQD were allowed with no smoking at all during Week 4. Participants who did not provide a CO reading at week 4 were considered to be smoking. Participants lost to follow-up were considered to be smoking. Abstinence at 12 weeks was defined as self-reported sustained abstinence since TQD (with up to 5 lapeses allowed) but it was not biochemically validated.

## Ethical approval

Ethical approval was obtained from the NHS National Research Ethics Service (Approval reference 10/H0709/85).

## Results

**Figure 1:**
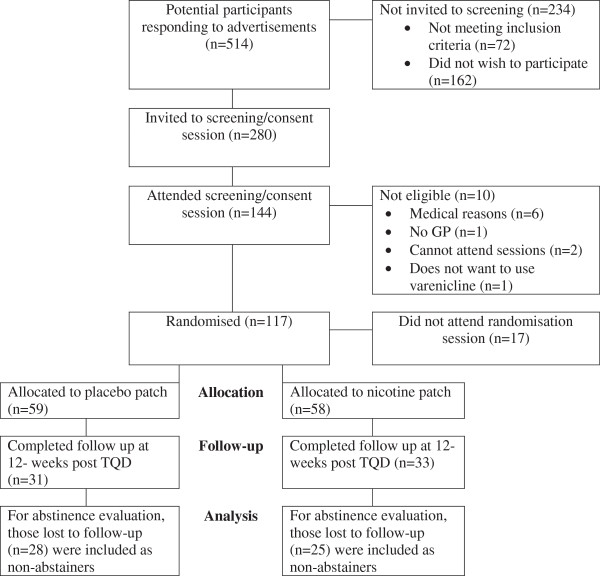
Flow of participants.

A total of 117 volunteers were enrolled and randomised. Figure 1 shows the flow of participants through the trial.

### Sample characteristics

Table 1 shows the baseline charateristics of the sample. There were no significant differences between the two study arms.

**Table 1:** Baseline characteristics of participants

	**Placebo patch** (N=53-59)*	**Nicotine patch** (N=56-58)*
Age (SD) in years	43.8 (11.0)	45.3 (10.8)
Cigarette consumption (SD)	17.4 (8.1)	18.7 (8.0)
Baseline CO (SD)	21 (10.4)	21 (8.5)
FTND (SD)	4.9 (2.6)	4.8 (2.2)
Age when started smoking (SD)	17.4 (6.8)	18.4 (5.5)
Number of previous quit attempts (SD)	2.8 (1.1)	2.9 (1.2)
Male N (%)	40 (68)	38 (66)
White British N (%)	36 (61)	37 (64)
Married N (%)	16 (27)	15 (26)
Left school at 16 N (%)	48 (41)	46 (40)
Hand-rolled cigs N (%)	12 (20)	11 (19)
Partner smokes N (%)	15 (25)	15 (26)
In paid employment N (%)	49/58* (85)	41 (71)
Body mass Index (SD)	26 (4.6)	27 (4.8)

* Ns differ due to missing data.

### Adherance to medications

Table 2 shows adherence to medication use at different time points. There was no difference between the two study arms at any time point. The adherence to medications during the crucial first week post-TQD was high. Eight participants did not provide data (6 in placebo and 2 in nicotine patch arm), among the rest only one participant was not using patches and all were using varenicline.

**Table 2:** Adherence to medications

	**Placebo patch**	**Nicotine patch**
Not using patch at 24 h	6/52	5/53
No use of patch in the past week at 1W post-TQD	0/53	1/56
No use of patch in the past week at 4W post-TQD	11/53	13/51
Not taken varenicline at 24 h	0/52	3/53
Not taken varenicline in the past week at 1W post-TQD	0/53	0/57
Not taken varenicline in the past week at 4W post-TQD	6/53	6/53
Not taken varenicline in the past week at 3M post-TQD	23/31	24/33

* N varies dues to missing data.

### Effect of varenicline and nicotine patch combination on withdrawal symptoms

A total of 85 participants (43 in placebo patch and 42 in nicotine patch arm) of 105 (52 in the placebo and 53 in the nicotine arm) who were successfully contacted by telephone at 24 hours after their TQD reported abstinence from smoking and provided MPSS data. The two groups did not differ in urges to smoke (2.8 vs 2.9, F=0.18, P=0.67) or in change from baseline in the composite symptoms ratings (0.14 vs 0.07, F=0.31, p=0.58). One week after the TQD, 33 (placebo patch) and 40 (nicotine patch) participants who were abstinent at that time-point provided MPSS ratings. There was no difference between the study arms in urges to smoke (2.7 vs 3.0, F= 2.14, P=0.15) or in the change in the composite score (0.42 vs 0.42, F=.001, P=0.98). At four weeks after TQD, 35 (placebo patch) and 35 (nicotine patch) of participants who achieved sustained abstinence provided MPSS data and 34 (placebo patch) and 35 (nicotine patch) provided urge ratings. There were no differences in urges to smoke (2.1 vs. 2.2, F=0.08, p=0.78) or in the change from baseline in the composite symptoms ratings (0.05 vs. 0.01, F=0.08, p=0.78).

Looking at each withdrawal symptom at each time point separately, there was a significant difference in the change in depression ratings between TQD and 24 hours post-TQD in 43 participants on placebo patch (−0.14) and 42 participants on nicotine patch (−0.38) (F=4.10, P=0.046). This however, was due to a higher depression rating in the nicotine patch arm at TQD (1.21 vs 1.43 in the placebo patch and nicotine patch arm, respectively), i.e. prior to the initiation of the patch treatment. At 24 hours after TQD the ratings of the two groups were similar (1.07 vs 1.05).

Among particiants abstinent for 4 weeks who provided weight data at baseline and at 4 weeks (N=35 in the placebo and N=35 in nicotine patch arms), the weight gain was 0.71 and 1.60 kg in the 2 groups respectively (F=4.26, p=0.04). One participant in the placebo arm lost 5.5 kg. Removing this outlier made the difference non-significant. The two groups did not differ in their ratings of hunger at 24 hours (0.17 vs 0.50 in the placebo and nicotine group, respectively, F=1.45, p=0.23); 1 week (0.53 vs 0.42, F=0.24, p=0.63) or 4 weeks post TQD (0.29 vs 0.21, F=0.1, p=0.74).

There were no differences or trends emerging for any other single withdrawal symptom at any time-point.

### Effect of varenicline and nicotine patch combination on abstinence rates

Table 3 shows the rates of continuous validated abstinence (not a puff since the TQD) at 24 hour and 1 week and the Russell Standard sustained validated abstinence at 4 weeks (up to 5 cigarettes allowed with no smoking during the previous week). There were no differences between the two study arms at any time point. Self-reported sustained abstinence rates at 12 weeks were 29% vs. 36% in the placebo and nicotine patch groups, respectively (*x*^2^ = 0.73; p = 0.39).

**Table 3:** Effect of varenicline + NRT combination on abstinence rates

**Period after TQD**	**Placebo patch**	**Nicotine patch**	**Pearson Chi-square; p value**
	**N=59**	**N=58**	
24 hours N (%)	47 (80)	46 (79)	*x*^2^ =.00; p = 0.96
1 week N (%)	35 (59)	40 (69)	*x*^2^ =1.18; p = 0.28
4 weeks N (%)	35 (59)	35 (60)	*x*^2^ =0.01; p = 0.91

### Varenicline non-responders

Patients who have a weak reaction to varenicline during an extended pre-quit period have been shown to have low success rates [5]. In theory, the addition of nicotine replacement could be of particular benefit to such patients. To test this hypothesis, we evaluated separately the effect of nicotine patches on participants who did not reduce their CO reading by 50% or more during their pre-TQD varenicline dosing.

There were 18 participants who reduced their CO reading by over 50% during the pre-quit varenicline use (varenicline reactors). Their sustained abstinence rate at 4 weeks was 78%, compared to 57% in non-reactors (*x*^2^ = 2.85; p=0.09). Looking at the sub sample of non-reactors only (49 in the placebo and 50 in the nicotine patch arm), the abstinence rates at 1 and 4 weeks after TQD were 55% and 66% (*x*^2^ = 1.23; p = 0.27) and 53% and 60% (*x*^2^ = 0.49; p = 0.49) in the placebo patch and nicotine patch arm, respectively. The non-reactors in the two study arms did not differ in urges to smoke or other withdrawal symptoms at 24 hours, 1 or 4 weeks after TQD.

### Adverse events

There were no differences in ratings of nausea between the two groups at any time point (1.6 vs 1.5, 1.7 vs 1.8 and 1.5 vs 1.4 at 24 hour, 1 week and 4 weeks after TQD in the placebo patch and nicotine patch arm, respectively, all NS). There was one serious adverse event in the placebo arm (musculoskeletal injury) that was unrelated to the study medication.

Table 4 shows other adverse events reported by more than 5% of participants.

**Table 4:** Adverse events reported by >5% of participants in at least one study arm

**AE reported**	**Number of participants experiencing the problem**		**Pearson Chi-square; p value**
	**Placebo patch** **N=59**	**Nicotine patch N=58**	
Abnormal dreams	5	12	*x*^2^ =3.5; p = 0.06
Headache	4	6	*x*^2^ =.48; p = 0.49
Insomnia	11	11	*x*^2^ =.00; p = 0.97
Nausea	26	33	*x*^2^ =1.93; p = 0.17

## Comment

Adding nicotine patches to varenicline had no beneficial or detrimental effect on urges to smoke, withdrawal discomfort, abstinence rates, or adverse effects profile.

Several issues need to be considered when interpreting these results.

The study had only a short-term follow-up. This however, would limit the generalisability of the results primarily if the results were positive. It is unlikely that a lack of effect during the acute withdrawal period when stop-smoking medications exert their main impact could change into a significant effect later on.

The sample size was sufficient to detect small differences in withdrawal ratings and craving and also a clinically meaningful difference in short-term abstinence rates. We cannot rule out a possibility of a subtle effect on abstinence rates detectable on a large sample. Such an effect seems unlikely though, because it would presumably be mediated by lowering of withdrawal discomfort and craving, and these parameters were not affected.

The negative results cannot be attributed to low compliance with medication use, as almost all participants used both medications during the first week after TQD when any beneficial effects would be expected to be the strongest. We used 16 h/15 mg patch which has extensive evidence of its efficacy [18]. The 24 hour patch has been shown to have stronger effects on morning urges to smoke [21], but both types of patches have the same effect on smoking cessation outcomes [1]. It could be argued that other short-acting NRT formulations, which can be used opportunistically could be more effective. This is possible, but any gains in potentially higher efficacy of short-acting NRT products are usually undermined by the fact that oral NRT products and nicotine nasal spray have more side effects and are less user-friendly then patches and generate lower adherence. Good sustained adherence to oral NRT products and nasal spray usually requires a period of supervised frequent and regular use [22]. Where the expectation is that the product will be used only occasionally, as a supplement to another treatment, the adherence is likely to be low. Nevertheless, our results should be generalised to alternative NRT products with caution. There also remains a possibility of type II error, i.e. that there is a difference but the trial did not detect it.

Adding nicotine patches to varenicline did not increase the incidence of nausea or of any other adverse events. There was a trend for the active patch group to report more abnormal dreams, but the results overall raised no concerns regarding the safety of combining the two treatments.

One possible interpretation of the lack of synergy between the two medications is that NRT and varenicline achieve their effects via similar target mechanisms, which overlap to a large or even full extent. Varenicline may act on a more limited range of nicotinic receptors than nicotine itself, but these seem to include those involved crucially in the rewarding effects of smoking. By blocking such receptors, varenicline may be limiting any potentially beneficial effects of NRT as well. E.g. nicotine patches normally alleviate weight gain in continuous abstainers [23] but they had no such effect here.

Nicotine patches did not improve outcomes significantly in the subgroup of smokers who did not have a strong response to varenicline early on. This finding is more tentative than the main result because there was a trend in favour of nicotine patches and the study was not powered for sub sample analyses. Whether or not any patch effects are blocked by varenicline more in varenicline responders than in non-responders, an interesting question arises as to whether varenicline non-responders may benefit from NRT in the absence of varenicline. If this were the case, treatment efficacy could be improved by switching smokers who show no reaction to varenicline during the pre-quit period over to NRT. Future studies should evaluate this notion further.

The trial results have a practical implication. Finding a positive effect would indicate that a large-scale study with a long-term follow-up is warranted, but it would not provide a definitive proof of efficacy. A negative result on the other hand provides an indication that such a trial is unlikely to yield strong positive results. There is a widespread interest in combining NRT and varenicline in the hope of improving treatment outcomes. The results of this study suggest that such practice may not be productive or economical, although further trials would be useful to exclude the possibility of type II error.

## Abbreviations

CO: Carbon monoxide; FTND: Fagerstrom test for nicotine dependence; nAChRs: Nicotinic acetylcholine receptors; NRT: Nicotine replacement therapy; SD: Standard deviation; MPSS: The mood and physical symptoms scale; TQD: Target quit day; MHRA: UK Medicines and healthcare products regulatory agency.

## Competing interests

PH and HM have received research funding from, and provided consultancy to manufacturers of smoking cessation medications.

## Authors’ contributions

PH, HM, KM and AD designed and conducted the study, interpreted the data, and wrote the manuscript. All authors made substantial contributions to conception and design, or acquisition of data, or analysis and interpretation of data; drafting the article or revising it critically for important intellectual content; and final approval of this version of the manuscript. All authors read and approved the final manuscript.

## Pre-publication history

The pre-publication history for this paper can be accessed here:

http://www.biomedcentral.com/1741-7015/11/140/prepub
